# Life on Railroads

**Published:** 1857-01

**Authors:** 


### LIFE ON RAILROADS.

NOTWITHSTANDING occasional calamities on railroads, that kind of travel is by very far safer than by carriage, sail, or steamship. The New Jersey Railroad, between New York and Philadelphia, by way of Trenton and Princeton, has transported twenty millions of persons, without the loss of a single passenger, *in his proper place*, during a period of twenty years' running, day and night.

The Macon and Augusta Railroad of Georgia, has also been in operation for twenty years, and in all that time but ONE passenger has lost his life by accident, on a line of one hundred and ninety-two miles in length. All honor to the directories, whose practical intelligence, and to the engineers and conductors, whose high competency and ceaseless care have contributed to such results. Intelligent, competent, and well-paid employées can almost annihilate railroad accidents. Where so much human life is at stake, fifty thousand in any single day, in our own country, public opinion should demand that such salaries should be given as would secure the services of men of education, of moral worth, and of social respectability, from the switchman up to the conductor, engineer, and superintendent.

During the first half of the year eighteen hundred and fifty-four, there was in Great Britain but one accident in every seven million of railroad passengers. From this statement, we are not certain that it is not safer to spend our time "on a rail" than on Broadway. It would have been of practical interest if, in the questions of the Secretary of the Treasury, as given in *Holley's New York Railroad Advocate*, (conducted with scientific ability,) there had been one inquiry—" What was the *position* occupied at the moment of the accident, by any passenger whose life was lost." Let the public press reiterate two facts.

First: Three-fourths of the lives lost on rail cars, in motion, are of passengers who *were not in their proper positions*—meaning thereby, that they should not only be in their seats, but keep their heads and limbs inside the cars. We have often admired the arrangements in the cars between New York and Philadelphia, in this respect. A passenger cannot put his head or arm outside without standing up or leaning forward in a painful position. Such a large proportion of people have so little common sense, or uncommon either, that the true policy is to put it out of their power to be hurt; and that can be done to a very great extent.

The *Second* item of railroad travel, which ought to be reiterated is—A train may go at the rate of thirty or forty miles an hour, with its five hundred passengers, be instantaneously arrested, the cars turned upside down, their seats shivered to atoms, and the flooring riven into myriads of splinters, every car filled with passengers—and not a single life lost, or limb broken, or other serious injury, to a passenger *in his place*.

The proof of this is—*we saw it*—the Express Train at *Yonkers*, about three years ago. *Cause*, the switchman, a common laborer, neglected his duty. In an instant he took to his heels—and, for aught we know, is running yet—for he has never been seen there since.

